# The evolution of PRRT for the treatment of neuroendocrine tumors; What comes next?

**DOI:** 10.3389/fendo.2022.941832

**Published:** 2022-10-31

**Authors:** Philip E. Harris, Konstantin Zhernosekov

**Affiliations:** ITM Oncologics GmbH, Garching, Germany

**Keywords:** peptide receptor radionuclide therapy (PRRT), targeted radionuclide therapy (TRT), neuroendocrine tumors (NETs), Lutetium-177, Ga-68 PET, dosimetry

## Abstract

Lu-177 has been developed for the treatment of patients with peptide receptor radionuclide therapy (PRRT). A second generation pure no-carrier-added Lu-177 has a high specific activity and has waste disposal advantages over the first generation carrier-added Lu-177. PRRT has recently been developed for the treatment of neuroendocrine tumors (NETs). The majority of pancreatic and gastroenteric NETs (GEP-NETs) express the somatostatin receptors (SSTRs) 2 and 5. These receptors can be specifically targeted with a somatostatin peptide analogue (DOTATOC/DOTATATE) which can be chelated to a positron emission tomography (PET) emitting radioisotope such as Ga-68 for imaging or to a β-emitting radioisotope Lu-177 for therapy. A key advantage of this approach is that the receptor expression can be demonstrated by PET imaging before the patient is treated. Clinical studies in G1 and G2 GEP-NETS have demonstrated that PRRT is extremely effective in terms of progression free survival (PFS), symptom control and quality of life, with a well-established safety profile. A beneficial effect on outcome survival awaits to be confirmed. The first commercially available product Lu-177-DOTATATE was approved following the NETTER-1 trial in G1 and G2 GE-NETS. Lu-177-DOTATATE 7,4 GBq every 8 weeks for 4 cycles, together with octreotide LAR 30 mg monthly, demonstrated a median PFS of 28,4 months compared to 8,5 months for octreotide LAR 60 mg monthly. A second pivotal study COMPETE is currently in progress, comparing no carrier-added (n.c.a.) Lu-177-DOTATOC to the m-TOR inhibitor Everolimus in both GE-NETs and PNETs. Two studies, NETTER-2 and COMPOSE are currently underway in patients with high grade G2 and G3 NETs. Novel SSTR antagonists are being developed as next generation targeting molecules for SSTR2-expressing tumors. Antagonists have a higher tumor binding to receptors than agonists, opening up the potential indications for SSTR2 targeting to tumors which have a relatively lower expression of SSTR2 compared to NET such as small cell lung cancer, hepatocellular carcinoma and breast cancer. In addition to Lu-177, radioisotopes with different radiation properties such as Tb-161 and the α-emitter Ac-225 are being developed which have the potential to improve treatment efficacy across the range of G1 to G3 NETs.

## Introduction

Diagnostic and therapeutic radionuclides offer an excellent platform for the development of innovative drugs, which enable non-invasive visualization of diseases and complementary targeted treatments. This innovation in nuclear medicine and an increasing demand for high quality radionuclides and radiopharmaceuticals has triggered the expansion of nuclear medicine as a hospital specialty, together with the development of a new radiotheranostics industry.

This review focuses on the development of radionuclides for PRRT/targeted radionucide therapy (TRT) from the radiochemical perspective, recent and key ongoing clinical studies and future perspectives in NET.

## Lutetium-177 – The gold standard for radionuclide treatment

After the introduction of suitable macrocyclic chelators into the targeting molecules, trivalent radiometals such as Yttrium-90 (pure high energy β^-^ -emitter), gained importance for the targeted therapeutic treatment of serious oncological disease (1) Lutetium-177, in particular, has demonstrated excellent physical properties which enables the precise delivery of cytotoxic doses of beta irradiation to small and large malignant lesions. Furthermore, by emitting soft beta radiation (E_β_ 0,134 MeV), Lutetium-177 radiolabeled compounds have a favorable safety profile, particularly in terms of nephrotoxicity. Small components of photons (112,9 keV, 6% and 208,4 kEv, 10%) enable the visualization and quantitative estimation (dosimetry) of biodistribution by means of single-photon emission computerized tomography (SPECT), without having a negative impact on safety (**Table 1**). Starting with Lutetium-177-based treatments of somatostatin receptor (SSTR) positive NETs in the late nineties, the use of the radionuclide has dramatically increased.

**Table 1:** Characteristics of industrially available therapeutic β^-^ -emitters.

Nuclide	T_1/2_	E_β_-_,av_ [MeV]	E_γ_ [keV](I_γ_[%])	Mean Tissue range [mm]	Imaging/Dosimetry	Specific activity/Radionuclide purity	Energy deposition
**^90^Y**	64.053 h	0.934	–	3.9	no	no-carrier-added/high purity	hard betas for large and heterogeneous tumors (min 5 mm lesions)
**^177^Lu**	6.647 d	0.134	112.9 (6) 208.4 (10)	0.67	*Good resolution*yes	1) carrier-added/low purity2) no-carrier-added/high purity	soft betas for small tumors (min 0.5 mm lesions)
**^131^I**	8.0252 d	0.182	284.3 (6) 364.5 (82) 636.9 (7)	0.91	*Low resolution* yes	no-carrier-added/high purity	large gamma-component might result in hematoxicity

An important aspect of the successful development of Lutetium-177-based therapies is the availability of the radionuclide with specific activity suitable for the radiolabeling of targeting molecules. The first generation carrier-added preparations of Lutetium-177 for radiolabeling became commercially available from the early 2000s. Lutetium-177 can be produced in a nuclear reactor by the irradiation of the highly enriched stable isotope Lutetium-176. A high cross-section for the neutron capture reaction enables reasonable specific activity to be achieved, although the final preparation still consists of a mixture of the stable and radioactive isotopes Lu-176 and Lu-177, respectively. The main drawback of this manufacturing pathway is the co-accumulation of long-lived metastable Lutetium-177m (half-life 160,44 d). Depending on the irradiation parameters the fraction of this long-lived radionuclidic impurity varies from 0,2 to 0,7%. Disposal of solid and especially liquid wastes, contaminated with the long-lived impurity, is costly and laborious.

A significant enhancement for the future development of targeted radionuclide therapies has been the implementation of the second generation no-carrier-added (n.c.a) Lutetium-177. The n.c.a. form results in significantly higher quality of the radioisotope – in particular very high specific activity (i.e. almost no “cold” atoms of the same element in the preparation) and high radionuclidic purity. These improved characteristics allowed broader implementation of Lutetium-177 into clinical routine as well as in radiopharmaceutical research.

No-carrier-added Lutetium-177 can be produced by utilization of an alternative, more elaborate manufacturing route. Neutron irradiation of highly enriched Ytterbium-176 with neutrons results in short-lived Yb-177, which decays to desired Lu-177 which is free from Lu-177m contamination. In order to provide the needs timely, research nuclear reactors supply Lu-177 world-wide.

An important characteristic of the no-carrier-added form is the fact that the quality does not depend on the performance among different nuclear reactors, with the highest level of specific activity being ensured for all radionuclide preparations. Furthermore, the specific activity of n.c.a Lutetium-177 remains high over the shelf-life of the product.

## Lutetium-177- DOTATATE/DOTATOC for treatment of Gastroenteropancreatic NETs 

Neuroendocrine neoplasms (NENs) are relatively rare neoplasms which may arise from pluripotential stem cells in addition to cells of the diffuse neuroendocrine system comprising the autonomic nervous system, the thyroid, lungs, pancreas and gastrointestinal tract (2). The classification of NENs has recently been updated by the WHO according to morphology and proliferation as well differentiated neuroendocrine tumors (NETs) (G1 to G3) and poorly differentiated neuroendocrine carcinomas (NECs) (G3) (**Table 2**) (3).

**Table 2:** WHO classification (2019) for gastroenteropancreatic NENs.

Morphology	Grade	Mitotic count (2mm^2^)	Ki-67 (%)
Well-differentiated NETs	G1	<2	<3
	G2	2 - 20	3 - 20
	G3	>20	>20
Poorly differentiated	G3	>20	>20
NECs • Small-cell • Large-cell			
MiNEN			
Tumor-like lesions			

MiNEN, mixed neuroendocrine/nonendocrine neoplasm; NEC, neuroendocrine.

carcinoma; NEN, neuroendocrine neoplasm; NET, neuroendocrine tumor.

WHO, World Health Organization.

NETs may be clinically silent (‘non-functioning’) or may synthesize and secrete a variety of peptides and neuroamines which lead to the development of characteristic clinical syndromes, including carcinoid syndrome and functional pancreatic tumors such as insulinoma (hypoglycemia) and gastrinoma (gastric hyperacidity). The non-functioning tumors tend to present late with widespread metastatic disease, particularly in the liver. NETss tend to be indolent in character and patients can still live for relatively prolonged periods with good quality of life even with metastatic disease (4).

The incidence of NENs is increasing year-on-year. In the USA, the SEER database has documented a 6,4-fold increase in incidence between 2007 (1,09 per 100.000) and 2012 (6,98 per 100.000) (5). This increase has included gastroenteropancreatic (GEP) NENs with the highest incidence being seen in adults aged 74-80) (6) The age-standardized incidence rate of NENs in the UK between 2013-2015 has been reported as 8.6 per 100,000, with an incidence of 4.6 per 100,000 for gastroenteropancreatic (GEP) NENs (7).

GEP-NETs are graded with increasing aggressivity as G1, G2 or G3, based on Ki-67 labeling index, mitotic count and histological differentiation (**Table 2**) (3). Well differentiated G1 and G2 GEP-NETs typically show a slower progression and better prognosis than G3 GEP-NETs (8). A characteristic feature of GEP-NETs is that a majority express functioning somatostatin receptors (SSTRs) 2 and 5. This renders the tumors amenable to treatment with synthetic somatostatin peptides, which can inhibit functional secretion and also stabilize tumor growth (9). These synthetic peptides, conjugated with a chelator, can be efficiently radiolabeled with photon-, positron- or particle-emitting radionuclides. The expression of SSTR2 enables GEP-NETs to be imaged with receptor targeted high resolution PET/CT (DOTATOC/DOTATATE-Ga-68). In addition, tumors can be treated with therapeutic Lu-177 DOTATOC/DOTATATE (10).

Patients with G1 and G2 GEP-NETS often present late, with metastatic disease. Until recently, somatostatin analogues and the molecularly targeted drugs Sunitinib andEverolimus as well as chemotherapy in PNETs have provided the mainstays of treatment (11). These treatments usually result in disease stabilization for a limited period of time. In the RADIANT-3 trial, Everolimus achieved a progression free survival (PFS) of 11 months in pancreatic NETS (PNETs) and a similar PFS was achieved in midgut and pulmonary NETs in the RADIANT-4 trial (12, 13).

PRRT has recently emerged as a novel treatment option for patients with G1 and G2 GEP-NETs. The first pivotal prospective clinical trial (NETTER-1) was in G1 and G2 gastroenteric (GE) NETs, comparing the radiolabeled peptide Lu-177-DOTATATE (Oxodreotide) 7,4 GBq every 8 weeks for 4 cycles plus octreotide LAR 30 mg 4 weekly with the somatostatin analogue Octreotide-LAR 60 mg 4 weekly (control group). The trial met the primary objective of significantly improved progression-free survival (PFS) for Lu-177-DOTATATE with a hazard ratio (HR) of 0,18 (95% CI 0,11–0,29; p<0,0001) and a median PFS of 28,4 months compared to 8,5 months for the control group (14). The NETTER-1 study led to the registration of Lu-177- DOTATATE in 2018.

Long-term follow-up with a median duration of more than 6,3 years in each group, demonstrated that overall survival (OS) did not differ significantly between the study groups (HR 0,84 [95% CI 0,60–1,17]; two-sided p=0,30); median OS was 48,0 months in the Lu-177-DOTATATE group and 36,3 months in the control group. The adjusted HR to account for the 36% of patients in the control group who subsequently received PRRT was 0,73 (95% CI 0,4-1,34), suggesting that crossover to PRRT contributed to the OS results in the control group, although other unidentified confounding factors inevitably influenced the assessment (14, 15). During long-term follow-up, treatment-related serious adverse events of grade 3 or worse were recorded in three of 111 (2,7%) patients in the ¹⁷⁷Lu-DOTATATE group. Two of 111 (1,8%) patients given ¹⁷⁷Lu-DOTATATE developed myelodysplastic syndrome, one of whom died 33 months after randomization (this person was the only reported ¹⁷⁷Lu-DOTATATE treatment-related death). No new cases of hematological malignancy were reported during long-term follow-up (15).

An important clinically significant effect was the improvement of overall health quality of life which was prolonged for 22,7 months in the ¹⁷⁷Lu-DOTATATE group. This included improvement in daily activities, role functioning (participation in employment and leisure). Furthermore, patients also demonstrated improvements in diarrhea (48%), fatigue (50%), treatment scale and body image (63%) endocrine scale (61%) and GI scale (60%) (16). Detailed analysis of the diaries of the patients indicated significant decrease in the mean number of symptomatic days with diarrhea, flushing and abdominal pain which are the major symptoms of the patients with NETs (17). In summary, NETTER-1 study demonstrated that the use of ¹⁷⁷Lu-DOTATATE improved PFS and quality of life (QoL) together with a well-disposed safety profile.

Another alternative radiolabeled synthetic somatostatin analogue DOTATOC (Edotreotide) has been developed for the imaging and treatment of GEP-NETs The *in vivo* pharmacokinetics of the Lu-177-labeled peptides DOTATATE, DOTANOC, and DOTATOC have been investigated in patients with GEP-NETs (18). This study demonstrated favorable pharmacokinetic properties of radiolabeled DOTATOC with a more rapid clearance from healthy organs compared to DOTATATE and DOTANOC, providing a high tumor to background ratio and hence a high targeted dose of radiation to the tumor.

The first systematic evaluation of treatment data with n.c.a. Lu-177-Edotreotide in patients with GEP-NETs was reported by Baum et al. (19). In this retrospective study, the efficacy and safety of treatment with Lu-177-Edotreotide were evaluated in 56 subjects with metastasized, progressive NET (50% gastroenteric, 27% pancreatic, 23% other primaries) who had not received previous PRRT treatment prior to a new diagnosis of progression. Subjects received on average 2,1 (range 1– 4) cycles of ^177^Lu-Edotreotide as the sole treatment, administered in median doses of 7,0 GBq, at approximately three-monthly treatment intervals. In the total population, median PFS and OS were 17,4 and 34,2 months, respectively. In the subjects who had received more than one cycle of treatment, median PFS was 32,0 months for all and 34,5 months for GEP-NETs, with median OS of 34,7 months for both groups. No serious adverse events were noted, with no evidence of renal toxicity. In addition, a long-term safety follow-up of patients included in the retrospective study showed no lasting relevant hematotoxic effects and no long-term renal toxicity for up to 6 years after the 1^st^ PRRT. At the present time, it is standard practice to provide renal protection with a 2,5% lysine/arginine infusion which is given concomitantly with the PRRT infusion (20).

Based on the data from the study of Baum et al. (19), a Phase-III pivotal clinical trial, COMPETE has been initiated. COMPETE is a prospective, randomized, open-label multi-center Phase III study to evaluate the safety and efficacy of n.c.a. Lu-177-Edotreotide in comparison to Everolimus in patients with G1 and G2 PNETs and GE NETs. The patients have progressive, SSTR positive disease on SSTR imaging. Uniquely, patients may be included as first-line therapy. There are 3 sub-studies which focus on Lu-177-Edotreotide dosimetry and pharmacokinetics. These sub-studies are of great importance in the development of a personalized, precision therapy approach to the management of patients with PRRT. In addition, Lu-177 is uniquely non-carrier-added (n.c.a.), which means that it is a pure radionuclide of high specific activity.

**Figure 1:**
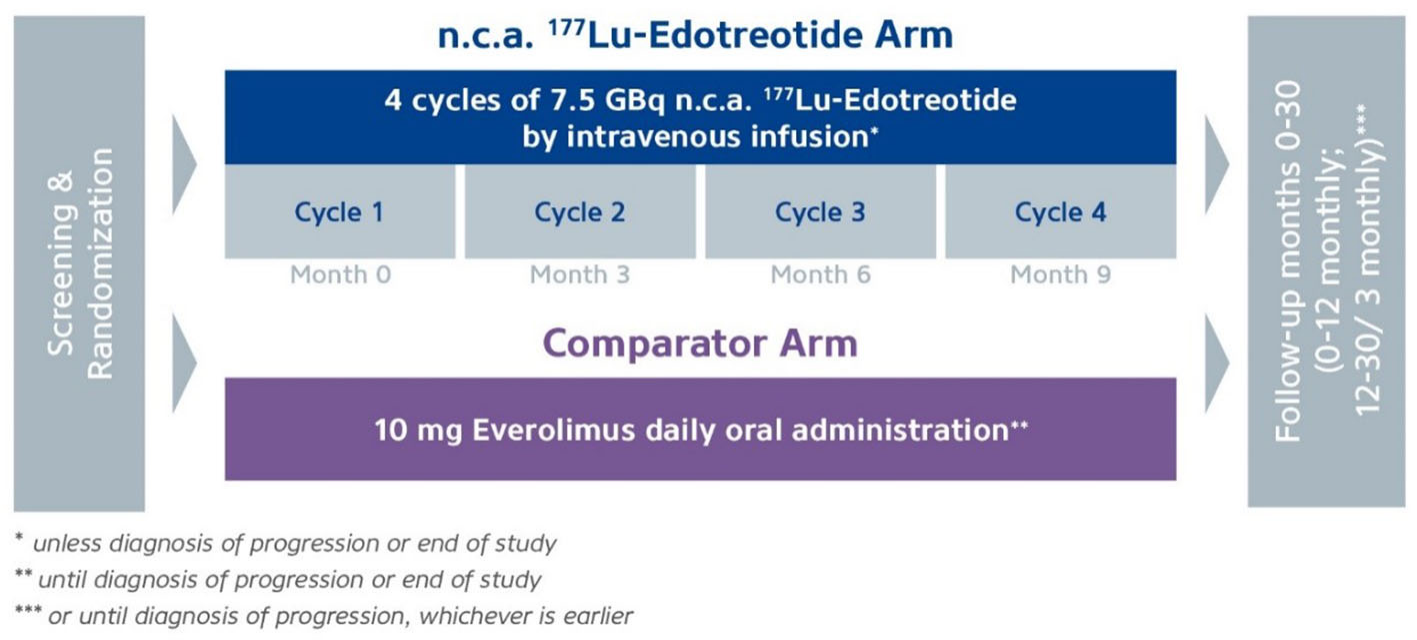
COMPETE study. Study subjects are randomised 2:1 between 4 x 12 weekly cycles of Lu-177-edotreotide vs 10 mg daily Everolimus, followed up for 30 months for PFS and 5 years for OS. *unless diagnosis of progression or end of study. **until diagnosis of progression or end of study. ***or until diagnosis of progression, whichever is earlier.

The study has completed the recruitment of 300 patients. A total of 200 patients will receive up to 4 cycles of ^177^Lu-edotreotide (7,5 GBq/cycle) every 3 months or until disease progression and 100 patients will receive Everolimus 10 mg daily for 24 months or until disease progression. The study duration is 30 months with 5 years follow-up for OS. The primary end-point is progression-free survival as assessed by RECIST 1.1. Key secondary end-points include safety and tolerability, dosimetry, objective response rate, overall survival and quality of life (**Figure 1**).

Patients with G3 NETs (**Table 2**) have more aggressive disease than the G1 and G2 NETs. A retrospective study of PRRT in G3 NENs has been reported by Zhang et al. (21). Sixty-nine patients were treated with either Lu-177 or Y-90-labelled somatostatin analogues (DOTATATE or DOTATOC). This was a heterogeneous group of patients both in terms of disease and treatment. Overall, the median PFS was 9,6 months and the median OS was 19,9 months. When the patients were sub-grouped into NETs with a Ki-67 index of ≤ 55%, the median PFS was 11 months and the OS 24 months. For NECs with a Ki-67 index of ≥ 55%, the median PFS was 4 months and the median OS was 7 months. In patients who had positive SSTR imaging but negative F-fluorodeoxyglucose (F-18-FDG) uptake, the prognosis was considerably improved. Other retrospective studies have also reported beneficial responses to PRRT in G3 NENs, particularly those with a Ki-67 index of ≤55% (22–24). These low grade G3 NETs are of particular interest for further clinical development. The high grade G3 NECs (Ki-67 ≥ 55%) respond relatively poorly to PRRT. These tumors might benefit from combination therapies, particularly with radiation sensitizers, immune-oncology agents and with DNA repair enzyme inhibitors (**Table 3**) (25–27).

**Table 3:** Ongoing NET trials with PRRT.

NCT	Protocol/Trial ID	Trial Title	Trial Phase
NCT03972488	NETTER-2	A phase III multi-center, randomized, open-label study to evaluate the efficacy and safety of lutathera in patients with grade 2 and grade 3 advanced GEP-NET	III
NCT02743741	NA	Lu-DOTATATE treatment in patients with 68Ga-DOTATATE somatostatin receptor positive neuroendocrine tumors	NA
NCT05153772	ALPHAMEDIX02	Targeted alpha-emitter therapy of PRRT naive neuroendocrine tumor patients	II
NCT03773133	NA	Evaluate the safety, tolerability, biodistribution and anti tumour activity of 177Lu-OPS201 with companion imaging 68Ga-OPS202 PET/CT in previously treated subjects with locally advanced or metastatic cancers expressing somatostatin receptor 2 (SSTR2) (SSTR2+)	II
NCT03457948	NA	Pembrolizumab with liver-directed or peptide receptor radionuclide therapy for neuroendocrine tumors and liver metastases	II
NCT02489604	LUNET	Peptide receptor radionuclide therapy (PRRT) with 177Lu-DOTATATE in advanced gastro-entero-pancreatic neuroendocrine tumors	II
NCT03454763	LUTHREE	Optimizing the interval between cycles of PRRT with 177Lu-DOTATATE in SSTR2 positive tumors	II
NCT04525638	NA	A clinical study to assess the combination of two drugs (177Lu-DOTATATE and Nivolumab) in neuroendocrine tumours	II
NCT02736448	Lu-Ca-S	177Lutethium - Peptide receptor radionuclide therapy (Lu-PRRT) plus Capecitabine versus Lu-PRRT in FDG positive, Gastro-entero-pancreatic neuroendocrine tumors	II
NCT04543955	NA	Telotristat with Lutathera in neuroendocrine tumors	II
NCT03466216	NA	Phase 1 study of alphamedix™ in adult subjects with SSTR (+) NET	I
NCT04234568	NA	Testing the addition of an anti-cancer drug, triapine, to the usual radiation-based treatment (Lutetium Lu-177 DOTATATE) for neuroendocrine tumors	I
NCT04086485	NA	Lu-177-DOTATATE (Lutathera) in combination with Olaparib in inoperable gastroenteropancreatic neuroendocrine tumors (GEP-NET)	I
NCT04750954	NA	Testing the addition of an anti-cancer drug, m3814 (Peposertib), to the usual radiation-based treatment (Lutetium Lu-177 DOTATATE) for neuroendocrine tumors	I

NA, not applicable.

**Figure 2:**
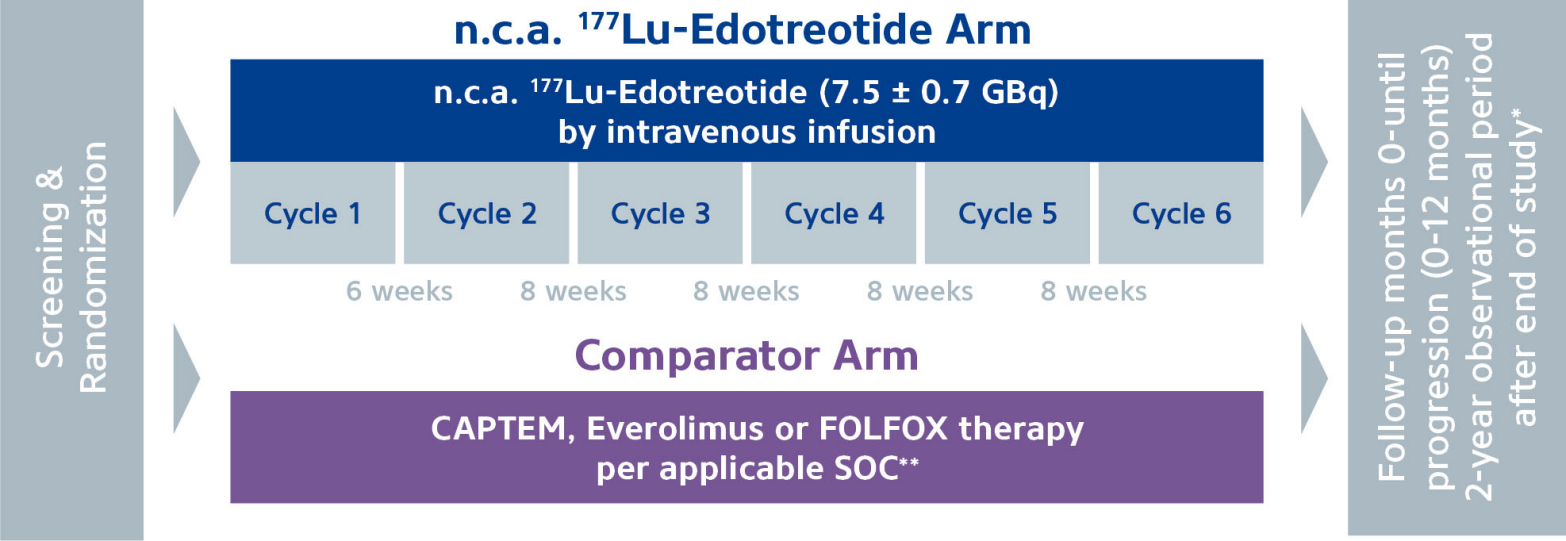
COMPOSE study. Patients are randomized 1:1 between 6 cycles Lu-177-edotreotide vs standard of care from CAPTEM, everolimus or FOLFOX therapy. Patients will be followed up for PFS and OS. *Survival data, information on further antineoplastic treatments and secondary malignancies. **Standard of Care.

There are two Phase 3 studies currently ongoing in high grade G2 and G3 NETs, NETTER-2 and COMPOSE. NETTER-2 is comparing Lu-177-DOTATATE as first-line therapy compared to 60 mg Octreotide LAR in patients with well-differentiated G2 or G3 GEP-NETs (Ki-67 index 10-55%). In this study, 222 patients are being randomized 2:1 to receive 4 cycles of Lu-177-DOTATATE (7,4 GBq 8 weekly) plus octreotide LAR 30 mg 8 weekly, with a continuation regiment of 30 mg 4 weekly, or octreotide LAR 60 mg 4 weekly. Patients who progress will have the option of cross-over or re-treatment, with a follow-up phase of 3 years. The primary endpoint is PFS, with OS, a secondary endpoint. In contrast, COMPOSE will compare first-line or second-line n.c.a. Lu-177-Edotreotide with best standard of care (SOC) in patients with well differentiated G2 and G3 GEPNETs (Ki-67 index 15-55%). A total of 202 patients will be randomized 1:1 to receive 6 cycles of n.c.a Lu-177-edotreotide (7,5 GBq) (**Figure 2**). The comparator arm comprises of a choice of SOC from Capecitabine and Temozolomide (CAPTEM), Everolimus or Folinic acid, Fluorouracil and Oxiplatin (FOLFOX). The treatment regimen is determined according to local prescribing information, until diagnosis of progression or end of the study. The primary endpoint is PFS with OS, a key secondary endpoint assessed up to 2 years after disease progression.

## Discussion

There are now extensive data available from retrospective studies and the more recent prospective studies attesting to the long-term safety of PRRT in NET. The main organs of concern are the bone marrow and the kidney. Transient hematotoxicity, particularly thrombocytopenia, is frequently seen usually around 4-6 weeks after a treatment cycle but this usually rapidly recovers, allowing a following cycle of treatment. Long-term myelodysplasia or leukaemia appears to occur in a little over 2% of patients, although historically, many have been heavily pre-treated. Renal toxicity, which is mitigated with concomitant amino acid infusion (20) has been mainly related to Y-90 therapy and is uncommon with Lu-177 (28, 29).

The diagnosis, management and follow-up of patients with GEP-NETs is detailed in recent ESMO Guidelines (3). PRRT has been shown to be benficial in the management of patients with symptoms of carcinoid syndrome (30). ESMO Guidelines recommend the use of Lu-177-DOTATATE following somatostatin analogue therapy, for the treatment of patients with carcinoid syndrome, diarrhea and/or flushing. PRRT is also recommended for patients with GE NETs who have progressive disease following treatment with somatostatin analogues or with everolimus and for PNETs following treatment with everolimus, sunitinib or chemotherapy (capecitabine and temozolomide/streptozotocin and 5-fluorouracil).

Although PRRT for the treatment of patients with NETs has provided a new paradigm for the management of patients with advanced metastatic disease, the optimal treatment regimen and also the positioning of PRRT in the treatment algorithm remain to be defined. At the present time, patients received a standard activity/dose of treatment at fixed intervals of 8 weeks (Lutathera) or 12 weeks (COMPETE study). The current treatment regimens are based on empirical observations in the clinic. Opportunities for further improving the management of patients are expected with new antagonist targeting molecules and the application of radionuclides which with different physicochemical properties to Lu-177. A number of clinical trials with PRRT in NETs are currently ongoing (**Table 3**).

### The future of targeted radiotherapy in NETS

At the present time, patients receive a standardized treatment regimen of ~7,5GBq 8-12 weekly for 4 cycles. There is a great deal of interest in individualizing patients’ therapy regimens based on dosimetry. All patients in the COMPETE study undergo dosimetric evaluation with 2D planar imaging at 0,5 hrs, 6 hrs, 24 hrs and 72-96 hrs after the first infusion and SPECT/CT for hybrid 2D/3D dosimetry at 24 hrs post each infusion. Sub-study A will compare the cumulative absorbed doses to kidneys and target lesions extrapolated from dose 1 to the cumulative absorbed dose from all 4 infusions. In sub-study B, the absorbed dose estimated by 3D dosimetry will be compared to estimates from planar 2D and hybrid 2D/3D dosimetry. It is expected that the dosimetry data from these studies will facilitate the application of dosimetry to individualized therapy, rather than the standard ‘one size fits all’ approach.

The currently available data for PRRT of NET patients are mainly for those with advanced disease, who have received previous therapies. These patients inevitably relapse at some stage. Earlier positioning in the treatment algorithm is of particular interest and data for the first-line therapy will be forthcoming from COMPETE, COMPOSE and NETTER-2 trials. Neoadjuvant therapy in PNETs is a subject of interest with anectodal reports of beneficial surgical outcomes in some patients (31).

It is apparent from dosimetry and safety that many patients are able to receive and benefit from additional cycles of treatment (32, 33). Combination therapies are also an area of interest particularly for patients with poorly differentiated tumors and a number of studies are ongoing. Retrospective evaluable data in 33 patients with advanced FDG positive NETS treated with capecitabine and Lu-177-DOTATATE have demonstrated partial responses in 10 patients (30%) and stable disease in 18 patients (55%), with a medial PFS of 31,4 months. Unsurprisingly, compared to monotherapy with PRRT in 37 patients there was a relatively high rate of G3 and G4 hematological toxicity of 16,2% (6 patients). Four patients (10,8%) stopped treatment after the first cycle due to toxicity (33).

Peptide antagonists targeted to SSTR2 have been shown to have a much higher binding to tumors than SSTR2 agonists and unlike agonists are not internalized. This has been demonstrated by the superior PET imaging of NETs with the antagonist Ga-68-NODAGA-JR11 compared to Ga-68-DOTATOC (34).

A first-in-human (FIH) dosimetry study of Lu-177-satoreotide teraxetan has been carried out in 20 patients with SSTR2 positive NETs (20% G1, 75% G2, 5% G3) who had been heavily pre-treated (35). Patients received a maximum activity of 7,4 GBq/cycle in 2 cycles. Although standard bone marrow dosing was followed, grade 4 hematologic toxicity occurred in 4/7 (57%) of patients after the second cycle. The protocol was then modified to limit the bone marrow dose to 1Gy and to reduce the activity of the second dose by 50%. The best OR was 45% (5% complete response (CR), 40% partial response (PR), 40% stable disease (SD), 15% progression (PD)). Median PFS was 21,0 months. These data demonstrate that although heavily pre-treated, conventional dosimetry parameters for the bone marrow used for agonist therapy cannot be used for antagonist therapy. Impressive efficacy was, however, seen in these patients with advanced disease.

Another FIH study with the peptide antagonist Lu-177-DOTA-LM3 (~6 GBq/cycle) has been performed in 51 heavily pre-treated patients, 35 of whom had previously received peptide receptor radiotherapy (36). Monitoring was achieved in 47 patients where a partial response was observed in 17 patients (36%), stable disease was observed in 23 patients (49%) and progressive disease was observed in 7 patients (15%). Of note, although all patients had positive PET scans with Ga-68-NODAGA-LM-3, 37 patients (73%) had negative PET scans with Ga-68-DOTATOC/DOTATATE, demonstrating the increased tumor binding with LM-3 compared to agonists. Unlike the first study, severe hematologic toxicity was not seen.

In a very recent study, combination of LM-3 with Tb-161 has been tested on mice xenografts (37). Tb-161 is known to emit the β^-^ and Auger electrons which are thought to be effective for the treatment of single cancer cells due to their high linear energy transfer. This combination therapy demonstrated impressive *in vitro* potency and *in vivo* efficacy, relative to Lu-177-DOTA-LM-3.

The increased tumor binding with antagonists opens up the potential indications for SSTR2 targeting to tumors which have a relatively lower expression of SSTR2 compared to NET such as small cell lung cancer, hepatocellular carcinoma and breast cancer. Combination with Tb-161 may further enhance the efficacy of antagonists in these conditions of very high unmet need.

## Conclusion

TRT to SSTR2 is a rapidly evolving field, particularly following NETTER-1, with the subsequent approval of Lu-177-DOTATATE for G1/G2 NET (14). In addition to GE NETs, the COMPETE study which compares n.c.a. Lu-177-DOTATOC with the approved comparator everolimus, will also provide novel prospective data for PNETs. A number of other clinical trials are also in progress which will expand the treatment options available and answer important questions such as dosimetry, the positioning of PRRT/TRT in the treatment algorithm, treatment cycles and extended therapy (**Table 3**). At the present time, n.c.a. Lu-177 is the standard radioisotope used in PRRT/TRT, although other radioisotopes, notably the alpha emitter Ac-225 and Tb-161 are also in development (**Table 3**). Beta emitters are ideally suited to the treatment of NETs, which are indolent growing tumors with a large metastatic bulk. Tb-161 may be particularly suited to non-internalizing antagonists, whereas more aggressive tumors may benefit from the low penetration but high energy alpha emitters, which result in highly localized cytotoxicity with double strand DNA breaks (38).

The field of targeted radiotheranostics although not new, is rapidly developing. The ability to deliver precise, highly targeted radiation therapy with superior efficacy and safety compared to other oncology treatments is setting a new standard in the therapeutic armamentarium for patients not only with NET but with other oncological disorders.

## Author contributions

PH and KZ devolped the idea and wrote the manuscript together. All authors contributed to the article and approved the submitted version.

## Conflict of interest

PH and KZ are employees of ITM Oncologics GmbH.

## Publisher’s note

All claims expressed in this article are solely those of the authors and do not necessarily represent those of their affiliated organizations, or those of the publisher, the editors and the reviewers. Any product that may be evaluated in this article, or claim that may be made by its manufacturer, is not guaranteed or endorsed by the publisher.
